# The impact of aging on innate and adaptive immunity in the human female genital tract

**DOI:** 10.1111/acel.13361

**Published:** 2021-05-05

**Authors:** Marta Rodriguez‐Garcia, Mickey V. Patel, Zheng Shen, Charles R. Wira

**Affiliations:** ^1^ Department of Immunology Tufts University School of Medicine Boston MA USA; ^2^ Department of Microbiology and Immunology Geisel School of Medicine at Dartmouth Lebanon NH USA

**Keywords:** Dendritic cells, epithelial cells, female reproductive tract, fibroblasts, menopause, resident memory T cells, sex hormones, sexually transmitted infections, TGFβ

## Abstract

Mucosal tissues in the human female reproductive tract (FRT) are primary sites for both gynecological cancers and infections by a spectrum of sexually transmitted pathogens, including human immunodeficiency virus (HIV), that compromise women's health. While the regulation of innate and adaptive immune protection in the FRT by hormonal cyclic changes across the menstrual cycle and pregnancy are being intensely studied, little to nothing is known about the alterations in mucosal immune protection that occur throughout the FRT as women age following menopause. The immune system in the FRT has two key functions: defense against pathogens and reproduction. After menopause, natural reproductive function ends, and therefore, two overlapping processes contribute to alterations in immune protection in aging women: menopause and immunosenescence. The goal of this review is to summarize the multiple immune changes that occur in the FRT with aging, including the impact on the function of epithelial cells, immune cells, and stromal fibroblasts. These studies indicate that major aspects of innate and adaptive immunity in the FRT are compromised in a site‐specific manner in the FRT as women age. Further, at some FRT sites, immunological compensation occurs. Overall, alterations in mucosal immune protection contribute to the increased risk of sexually transmitted infections (STI), urogenital infections, and gynecological cancers. Further studies are essential to provide a foundation for the development of novel therapeutic interventions to restore immune protection and reverse conditions that threaten women's lives as they age.

## Graphical abstract

A review of immune changes in the female reproductive tract of women after menopause reveals that while there is a general decline in immune protection, specific immune aspects are upregulated or remain unchanged in a compartmentalized manner. Understanding how immune protection changes in the reproductive tract in the years following menopause is essential to protect women from sexually transmitted infections and gynecological cancers as they age.

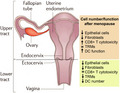

## 1 INTRODUCTION: CHANGES IN SUSCEPTIBILITY TO GENITAL INFECTIONS AND GYNECOLOGICAL CANCER DUE TO AGING

The aged population (>60 years old) is increasing rapidly and projected to grow to 1.4 billion by 2030, with women accounting for approximately 2/3 of individuals in this age group (He et al., 2016). With age, genitourinary infections and gynecological cancers increase, with profound effects on the morbidity and mortality of women (Gavazzi & Krause, 2002). Epidemiological studies show that urinary tract infections (UTI) and sexually transmitted infection (STI) rates increase in older women (CDC, 2016a, 2016b), presenting a public health challenge that must be addressed. The incidence of STIs has increased by 38% since 2010 in the 50–70 year age group (CDC, 2016a, 2016b). UTIs are often caused by *Escherichia coli* (Hu et al., 2004), which colonize the FRT in older women prior to spreading to the urinary tract (Ghosh et al., 2014; Hummelen et al., 2011). Sexual activity is a risk factor for STIs and some UTIs, the prevalence of which is not widely recognized in older adults (CDC, 2016a; Hu et al., 2004; Taylor et al., 2017).

In addition to genitourinary infections, aged women have a high burden of comorbidities associated with endometrial, ovarian, and cervical cancers (CDC, 2019). Uterine cancer is the most common gynecological cancer worldwide and the sixth most common cause of cancer death which occurs primarily in postmenopausal women, with an average age of diagnosis of 60 years (Henley et al., 2020; Lu & Broaddus, 2020). Accompanying this is an increase in human papillomavirus (HPV) (types 16 and 18), the underlying cause of cervical cancer and precancerous lesions (Chan et al., 2019; Gonzalez et al., 2010; Gravitt et al., 2013; Rositch et al., 2012). Despite the burden of STIs and gynecological cancer in older women, they are not recognized as a clinical priority. Aged women are also generally excluded from STI prevention trials (Herrera et al., 2010), vaccination recommendations, and prevention advice (Granville & Pregler, 2018). Thus, there is a critical need to understand how, as women age, immune protection against STIs and cancer changes in the FRT—the primary mucosal surface where pathology initiates.

## 2 UNIQUENESS OF THE AGING PROCESS IN THE FRT: MENOPAUSE AND AGING IN WOMEN

The aging process in women is accompanied by the transition into menopause. Menopause marks the end of natural reproductive potential with the permanent secession of menstrual cycles, caused by the decline in ovarian sex hormone production (estradiol and progesterone) (Maruoka et al., 2014). Since the average age at menopause is 50 years (Palacios et al., 2010), and the average life expectancy of women in the USA is 78 years, women live for 30–40 years in a postmenopausal environment with low concentrations of sex hormones. How this hormone‐deprived environment affects immune function overtime is of great importance in understanding the mechanisms involved in immune protection in older women. Importantly, long‐term survival after menopause cannot be fully reproduced in animal models (Walker & Herndon, 2008), highlighting the importance of studying aging effects with human samples.

**FIGURE 1:**
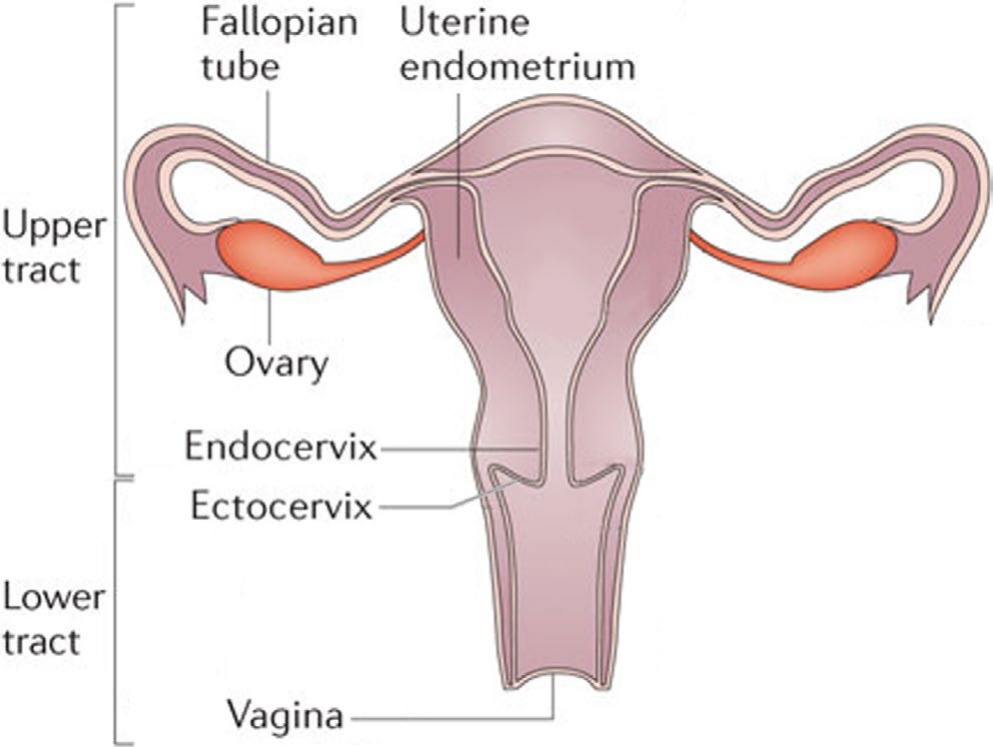
Diagram of the human female reproductive tract (FRT) showing the major tissue compartments. The upper FRT includes the fallopian tubes, endometrium, and endocervix, which are lined with columnar epithelial cells. The lower FRT consists of the ectocervix and vagina which is lined with squamous epithelial cells. The reproductive and immunological functions of each site are separate and distinct. Each site functions to optimize conditions for successful fertilization and implantation while protecting against sexually transmitted pathogens. Adapted from (Wira et al., 2015)

Not widely appreciated is that the immune system in the FRT is critical for reproductive success. Sex hormones tightly regulate immune function in the premenopausal FRT to ensure the balance between optimal conditions for pregnancy and protection against pathogens (Wira et al., 2015). To achieve this necessary balance, the FRT has evolved with distinct anatomical compartments consisting of the fallopian tubes, uterus (endometrium), endo‐ and ectocervix, and vagina (Figure 1). As reviewed elsewhere (Wira et al., 2015), each compartment contains adaptive and innate immune cells, but each site is separate and distinct regarding reproductive function and immune protection prior to menopause. Following menopause, immune cell populations and responses are dramatically altered. As women age, two interrelated processes overlap and contribute to changes in immune protection in the FRT: menopause and immunosenescence. While much is known about the effects of sex hormones on immune function in the FRT during the menstrual cycle, relatively little is known about the immunosenescent changes that occur after menopause and in the years that follow.

In this review, we consider immunosenescence throughout the FRT. We focus on changes in mucosal immune function following menopause and how they relate to potential changes on susceptibility to infections and the risk of gynecological cancers. Beyond the scope of this review are age‐related changes in the ovary, vulva, and other anatomically proximal organs, such as bladder and rectum, which contribute to morbidity in older women. Overall, following menopause, a growing body of evidence indicates that aging significantly alters adaptive and innate immunity, in ways that are distinct and site‐specific throughout the FRT.

## 3 CHANGES IN EPITHELIAL CELLS AND BARRIER PROTECTION INDUCED BY AGING

Epithelial cells line the surface of the FRT and are the first line of defense against incoming pathogens. They contribute to immune protection by (a) providing a physical barrier that separates the internal and external environments; (b) providing a chemical barrier composed of mucus, antimicrobials, cytokines, and chemokines that directly interact with pathogens and modulate the local immune system; and (c) mounting rapid innate immune responses to pathogens via pattern recognition receptors (PRRs). Little is known about how FRT epithelial functions change with age in postmenopausal women, since most studies focus on younger reproductive‐aged women.

### 3.1 Barrier protection

Epithelial cells form a physical barrier that protects underlying FRT tissues and its resident immune cells against potential pathogens and injuries. Epithelial cell phenotype varies with anatomical location in the FRT. The stratified squamous epithelium of the lower FRT (ectocervix and vagina) is 25–50 layers thick with superficial, parabasal, and basal layers (Patton et al., 2000). In contrast, the upper FRT (endocervix, endometrium, and fallopian tubes) is covered by a single layer of columnar epithelial cells. Whether increased barrier thickness correlates with increased protection against pathogens in the lower FRT is unclear. However, cervical ectopy, where columnar epithelium of the endocervix extrudes onto the surface of the ectocervix, is associated with increased transmission risk of HIV (Moss et al., 1991), human papillomavirus (HPV) (Rocha‐Zavaleta et al., 2004), C*hlamydia trachomatis* (Lee et al., 2006), and cytomegalovirus (CMV) (Critchlow et al., 1995).

Epithelial atrophy is common following menopause (Anderson et al., 1989; Farage & Maibach, 2006; Losif & Bekassy, 1984). Postmenopausal women have a thinner vaginal epithelium (21.4 vs 10.7 cell layers) compared to premenopausal women (Thurman et al., 2017), suggesting decreased barrier protection in the lower FRT. There is also a loss of hydration which leads to increased vaginal dryness, irritation, and inflammation. Loss of natural lubrication can lead to epithelial damage during sexual intercourse, potentially increasing pathogen access to the underlying tissue. Furthermore, epithelial wound healing is compromised following menopause in animal models (Ben Menachem‐Zidon et al., 2020; Shveiky et al., 2020), at other mucosal sites (Engeland et al., 2009; Horng et al., 2017) and in cell culture (Patel, M. Unpublished).

**FIGURE 2:**
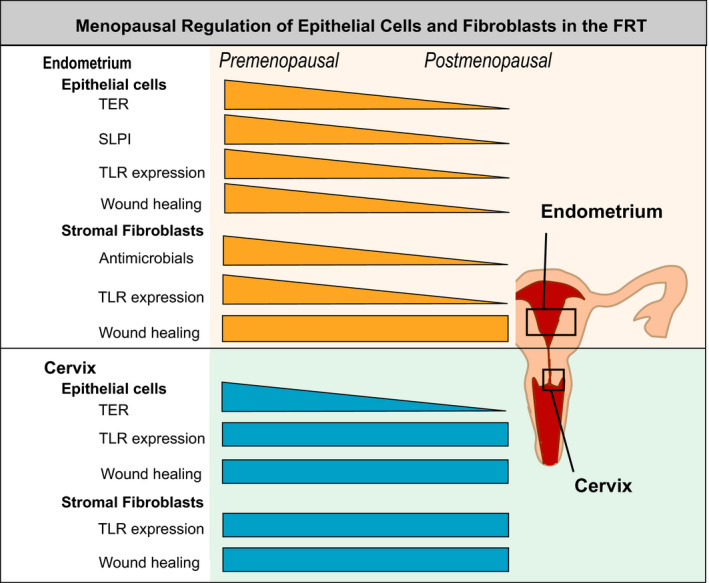
Regulation of epithelial cell and fibroblast function by menopausal status. This diagram shows key epithelial and stromal fibroblast functions and how they are modified after menopause. Triangles indicate a decline in cell function with menopause. A rectangle indicates no change following menopause. Effects are shown for the endometrium on the upper part and for the cervix (endocervix and ectocervix) on the lower part of the figure

Tight junction and adherens junction protein complexes link adjacent epithelial cells and serve as a selectively permeable barrier that allows movement of proteins and solute across the epithelium (Anderson & Van Itallie, 2009; Blaskewicz et al., 2011). Tight junctions are primarily composed of ZO‐1, occludin, and multiple claudin proteins, while adherens junctions are composed of N‐, P‐ and O‐cadherin. Tight junctions are precisely regulated throughout the menstrual cycle by sex hormones in premenopausal women (Fahey et al., 2006; Gorodeski, 2001a, 2007; Gorodeski et al., 2005; Iwanaga et al., 1985; Murphy et al., 1992; Zeng et al., 2004). While the effect of aging on tight and adherens junction expression in the FRT is relatively unknown, vaginal epithelial E‐cadherin levels are lower in postmenopausal women than premenopausal women (Thurman et al., 2017). Furthermore, levels of paracellular permeability and transcellular resistance are lower in ectocervical cultures from postmenopausal women compared to premenopausal women (Gorodeski, 2001b). Since pathogens such as HIV can also decrease tight junction integrity between endometrial epithelial cells (Mukura et al., 2017; Nazli et al., 2010), aging may exacerbate the movement of pathogens into the underlying tissue. Thus, aging potentially leads to an overall decrease in FRT epithelial barrier protection (Figure 2).

### 3.2 Pattern Recognition Receptors (PRRs)

PRRs are essential for the recognition and response to pathogens. PRRs include Toll‐like receptors (TLR) and retinoic acid inducible gene (RIG)‐like receptors (RLR), which recognize conserved moieties known as pathogen‐associated molecular patterns (PAMPs) characteristic of broad classes of pathogens. PRR expression varies within the FRT by anatomical location and cell type (Pioli et al., 2004; Zarember & Godowski, 2002). Human endometrial epithelial cells express TLRs1‐9, with TLR1, 2, 3, and 5 being expressed at the highest levels (Schaefer et al., 2005). Vaginal epithelial cells express TLR1, 3, 5, and 6, but not TLR4 (Fichorova et al., 2002). At the tissue level, TLR4, RIG‐I, MDA5, NOD1, and NOD2 expressions are highest in the upper FRT and decline in the lower FRT (Ghosh et al., 2012; Pioli et al., 2004). TLR2 expression is highest in the fallopian tubes and cervix but lowest in the endometrium and ectocervix. In contrast, TLR7, 8, and 9 are consistently expressed from the fallopian tubes to ectocervix (Hart et al., 2009).

TLR expression varies with menstrual cycle stage and is lower in endometrial tissues at the proliferative phase compared to the secretory phase. Endometrial epithelial immune responses are partially regulated by sex hormones. Estradiol decreases secretion of IL‐6, IL‐8, and MIF by endometrial epithelial cells in response to TLR3 or TLR4 stimulation (Fahey et al., 2008; Lesmeister et al., 2005). How the decline in ovarian sex hormones affects epithelial innate immune responses to PRR ligands is unclear. In preliminary studies with endometrial epithelial cells, we discovered a trend toward decreased TLR3 expression in older women (>75 years) compared to younger women (50–59 years) (Figure 2). Since exposure to the TLR3 agonist poly(I:C) induces a proinflammatory antiviral response in endometrial and vaginal epithelial cells (Patel et al., 2012, 2018a; Schaefer et al., 2005; Trifonova et al., 2009), decreased responsiveness to viral pathogens could compromise epithelial cell‐mediated innate protection throughout the FRT. Whether PRR expression in general and responsiveness to TLR agonists decreases with age in the FRT is unknown. At other sites in the body, increased age is associated with decreased responsiveness to PRR stimulation (Dunston & Griffiths, 2010; Iram et al., 2012; Panda et al., 1950).

### 3.3 Mucus

FRT epithelial cells produce a protective mucus layer that reduces direct contact with pathogens, such as HIV, by trapping them and preventing access to the epithelium (Lai et al., 2009; Shukair et al., 2013). Major constituents of mucus are the negatively charged glycoproteins known as mucins which form a network of protein complexes that can bind incoming pathogens. In premenopausal women, mucin (MUC) gene expression varies with menstrual status leading to changes in the overall properties of mucus including consistency and permeability (Elstein, 1978; Gipson et al., 1997; Vigil et al., 2009). Following menopause, the expression of vaginal MUC4 and MUC5AC decreases (Moncla et al., 2016) potentially reducing mucus binding capacity and its ability to interact with pathogens.

In the lower FRT of premenopausal women, vaginal mucus has an acidic pH that reduces HIV infectivity (Tyssen et al., 2018). Some studies show that postmenopausal women have increased vaginal pH compared to premenopausal women (Thurman et al., 2017), while others show no difference in pH between the two populations (Murphy et al., 2019). Changes in pH could be mediated via altered composition of the vaginal microbiome in older women that in turn increases vaginal pH (Murphy et al., 2019).

### 3.4 Antimicrobials and cytokines

Antimicrobials and cytokines secreted by epithelial cells are key protective components of cervical‐vaginal fluids which bathe the entire FRT. Antimicrobials interact with pathogens both prior to contact with the epithelium and within the sub‐epithelial stromal compartment (Fahey et al., 2005; Wira et al., 2010). FRT epithelial cells secrete multiple antimicrobials and cytokines including human β‐defensins (HBDs), SLPI, lysozyme, tracheal antimicrobial peptide, TNFα, IL‐8, CCL20, elafin, and cathelicidin (Fahey et al., 2008; Schaefer et al., 2005; Wira et al., 2010, 2011). These have potent antiviral, antibacterial, and anti‐fungal activity and represent an immunological barrier that protects epithelial cells and other cells in the underlying stroma. Sex hormones directly regulate the secretion of antimicrobials by epithelial cells *in vitro*. Estradiol stimulates the secretion of SLPI, elafin, and HBD2 by endometrial epithelial cells (Fahey et al., 2008), but inhibits HBD2 and elafin secretion by vaginal epithelial cells (Patel et al., 2013). We showed that, due to the absence of SLPI secretion, apical secretions by endometrial epithelial cells *in vitro* from postmenopausal women are unable to inhibit *Staphylococcus aureus* growth in culture in contrast to those from premenopausal women (Fahey & Wira, 2002). Loss of antibacterial activity via decreased expression of epithelial antimicrobials could be one mechanism by which older women become more susceptible to bacterial infections.

Several studies have investigated how sex hormones affect antimicrobial and cytokine secretions in the FRT (Cortez et al., 2014; Fahey et al., 2008; Patel et al., 2014; Wira et al., 2010) using cervical‐vaginal lavage (CVL) fluid that consists of the combined secretions of epithelial cells and immune cells from the upper and lower FRT. We and others have found changes that correlate with stage of the menstrual cycle (Keller et al., 2007; Wira et al., 2010). At midcycle (days 13–14), IL‐8, Surfactant Protein A, SLPI, HBD2, α‐defensins 1–3, and lactoferrin in cervical‐vaginal lavage (CVL) fluids are depressed and remain so for 7–10 days (Keller et al., 2007). In contrast, total protein levels and TGFβ remained unchanged during this time. Similarly, Cortez et al. (Cortez et al., 2014) demonstrated that IL‐6, MIP1α, MIP1β, TNFα, GMCSF, IFNα2, and IL‐10 all decreased at midcycle compared to the proliferative and secretory phases.

Changes in the antimicrobial and cytokine profile in CVL after menopause remain unclear. Decreased levels of TNFα (Jais et al., 2016; Thurman et al., 2017), CCL20 (Ghosh et al., 2019; Jais et al., 2016), SLPI (Ghosh et al., 2019; Jais et al., 2016; Murphy et al., 2019; Thurman et al., 2017), and HBD2 (Ghosh et al., 2019; Jais et al., 2016; Murphy et al., 2019; Thurman et al., 2017) were observed in multiple CVL studies comparing pre‐ and postmenopausal women. Despite these differences in antimicrobial and cytokine levels, there were no differences in anti‐HSV‐2 activity between pre‐ and postmenopausal women (Chappell et al., 2015; Thurman et al., 2017). Intriguingly, several studies showed increased anti‐HIV activity in postmenopausal CVL (Jais et al., 2016; Murphy et al., 2019), while others showed no effect (Ghosh et al., 2019; Thurman et al., 2017). Similarly, there was no difference in *Escherichia coli* inhibition (Murphy et al., 2019; Thurman et al., 2017). Together, these conflicting studies demonstrate the complexity of epithelial‐mediated immune protection in the FRT and suggest that the mechanisms of immune protection against incoming pathogens vary considerably between pre‐ and postmenopausal women.

## 4 CHANGES IN FIBROBLASTS INDUCED BY AGING

Fibroblasts form a dense layer of cells in the sub‐epithelial stroma where they surround local immune cells. While primarily considered structural cells, they are key players in the innate immune response within the FRT (Wira et al., 2015). Fibroblasts express multiple PRRs, including TLR2, TLR3, TLR4, TLR5, TLR6, TLR9, RIG‐I, and MDA5, with endometrium fibroblasts generally expressing higher PRR levels than other sites in the FRT (Patel et al., 2018b; Patel Shen, & Wira, 2018). They mount innate immune responses against a broad range of incoming pathogens characterized by increased secretion of inflammatory cytokines and chemokines, leading to increased chemotaxis of immune cells (Patel et al., 2018b; Patel Shen, & Wira, 2018). FRT fibroblasts secrete Type I and Type III interferons (IFNs) and thus induce an antiviral state in adjacent cells (Patel et al., 2018b; Patel Shen, & Wira, 2018). Secretions from fibroblasts can also directly inhibit pathogen survival. For example, ovarian and endometrial fibroblast secretions can inhibit HIV infection of target cells (Patel et al., 2018b; Patel Shen, & Wira, 2018).

Similar to other FRT cells, fibroblasts are sensitive to the presence of sex hormones, particularly in the endometrium. In premenopausal women, endometrial fibroblasts decidualize during the secretory phase of the menstrual cycle due to increasing levels of progesterone. *In vitro*, estradiol stimulates the secretion of hepatocyte growth factor (HGF) and stromal‐derived factor‐1 (SDF‐1α) (Coleman et al., 2009, 2012) and potentiates the upregulation of IL‐27 in response to TLR3 stimulation (Patel et al., 2018a).

The phenotypic and functional changes that FRT fibroblasts undergo following menopause with reduced exposure to sex hormones, and subsequent aging are relatively unknown. Sensitivity to sex hormones is retained in postmenopausal fibroblasts suggesting that exogenous hormones can modulate the function of FRT fibroblasts as women age (Gibson et al., 2018). Endometrial fibroblasts from perimenopausal women have an altered transcriptome compared to premenopausal women characterized by changes in expression for cytoskeleton, proliferation, and survival genes (Erikson et al., 2017). However, studies with other tissues such as the skin demonstrate that with increased age, fibroblasts undergo senescence (Wang & Dreesen, 2018), reduced proliferative capacity (Bentov et al., 2014), decreased wound healing (Mahmoudi et al., 2019), transition to an activated inflammatory phenotype (Wolf et al., 2012), and promote epithelial growth and tumor development (Krtolica et al., 2001) (Figure 2).

## 5 CHANGES IN T‐CELL DISTRIBUTION AND FUNCTION IN THE FRT

T cells are the most abundant leukocytes in the FRT of pre‐ and postmenopausal women (Givan et al., 1997; Rodriguez‐Garcia et al., 2014; Wira et al., 2015). However, after menopause, T‐cell populations undergo changes in distribution, phenotype, and function in a site‐specific manner.

### 5.1 CD4+ T cells

More than 95% of CD4+ T cells in the FRT have a memory phenotype and can be found scattered throughout the FRT (Saba et al., 2010; Yeaman et al., 2001). CD4+ T cells recognize peptides presented on MHC class II molecules on antigen presenting cells and play a major role in regulating adaptive immune responses. CD4+ T cells represent 35%–50% of CD3+ T cells in the FRT and, after menopause, CD4+ T‐cell presence is significantly reduced in the endometrium compared to the endocervix and ectocervix (Rodriguez‐Garcia et al., 2014). Within the CD4+ T‐cell population, menopause alters Th17 cell distribution of in the FRT. In premenopausal women, Th17 cells represent a major proportion of the total CD4+ T‐cell population in the endocervix and ectocervix, but are a minor fraction in the endometrium (Joag et al., 2015; Ma et al., 2020; McKinnon et al., 2011; Rodriguez‐Garcia et al., 2014). After menopause, Th17 cells significantly increase in the endometrium, without changes in endocervix and ectocervix (Rodriguez‐Garcia et al., 2014). This compartmentalization of Th17 cell distribution may be relevant for reproductive success, given the studies indicating that Th17 cells in human blood and animal models correlate with early pregnancy loss (Abdolmohammadi Vahid et al., 2019; Fu et al., 2014; Lee et al., 2012; Wang et al., 2020). In addition, Th17 cells play a central role in maintenance of epithelial barrier function and protection against extracellular bacteria and fungi (Sandquist & Kolls, 2018; Stockinger & Omenetti, 2017). Changes in FRT Th17 cell distribution and function with aging, and the potential consequences for immune protection remain unknown.

Another significant change after menopause is the increased expression of CCR5 on FRT CD4+ T cells. Increased CCR5 expression has been demonstrated on CD4+ T cells from the cervix and endometrium, with CCR5 preferentially expressed on Th17 cells (Meditz et al., 2012; Rodriguez‐Garcia et al., 2014; Trifonova et al., 2014). CCR5 is a chemokine receptor with important roles in reproductive function and also the coreceptor used by HIV to infect genital tissues (Saba et al., 2010), representing a marker for susceptibility to HIV acquisition. Increased susceptibility to HIV infection in postmenopausal women has been demonstrated in epidemiological studies analyzing sero‐discordant couples and in *ex vivo* HIV infection studies using tissue explants (European Study Group on Heterosexual Transmission of HIV, 1992; Rollenhagen & Asin, 2011; Thurman et al., 2017).

### 5.2 Tissue‐resident memory T cells

Particularly relevant for mucosal surfaces is the presence of tissue‐resident memory T cells (TRMs), which remain in tissues without recirculating, thereby providing first line local defense against reinfection and reactivation (Masopust & Soerens, 2019). TRMs have been involved in protection against genital infections, such as HSV‐2 and HPV, and cancer in animal models (Cuburu et al., 2012; Shin & Iwasaki, 2012; Shin et al., 2016). TRMs can be identified by CD69 and CD103 expression (Gebhardt et al., 2009; Mueller & Mackay, 2016). We and others have demonstrated that a high proportion of human FRT T cells express the tissue residency markers CD69+ and CD103+ (Cantero‐Perez et al., 2019; Duluc et al., 2013; Joag et al., 2015; Ma et al., 2020; Moylan et al., 2016; Oja et al., 2017; Rodriguez‐Garcia et al., ,^2017^, ^2018^, ^2020^). TRMs remain constant throughout the life span in multiple organs and mucosal surfaces (Thome et al., 2014). However, in the FRT, CD103+ T‐cell presence significantly changes with menopause and aging in a site‐specific manner (Rodriguez‐Garcia et al., 2018). Endometrial CD103+ T cells increase after menopause and remain constant with postmenopausal aging. In contrast, in the cervix, CD103+ T cells progressively decline after menopause as women age. CD103 can be expressed on CD4+ and CD8+ T cells (Rosato et al., 2017); however, age‐related changes in the FRT are specific to CD8+ CD103+ T cells, with no modifications on CD4+ CD103+ T cells, which represent less than 10% of the CD103+ T‐cell population (Rodriguez‐Garcia et al., 2018).

### 5.3 CD8+ T cells

The proportion of CD8+ T cells increases in the endometrium after menopause (Rodriguez‐Garcia et al., 2014; Trifonova et al., 2014), accompanied by modifications in distribution, phenotype, and function. Cytotoxicity is a key function of CD8+ T cells to eliminate infected and cancerous cells. Additionally, uniquely important to the FRT, CD8+ T cells mediate allogeneic rejection, which results in infertility (Erlebacher, 2013a, 2013b). To prevent rejection of the semi‐allogeneic blastocyst, still unknown mechanisms control T‐cell function specifically in the endometrium to suppress CD8+ T‐cell cytotoxic activity. Cytotoxic activity of endometrial CD8+T cells, including direct killing of allogeneic target cells, is significantly suppressed in premenopausal women compared to postmenopausal women (Rodriguez‐Garcia et al., 2020; White et al., 1997). Within premenopausal women, cytotoxic activity was further suppressed during the secretory phase of the menstrual cycle, when implantation and pregnancy is likely to occur in the endometrium (Rodriguez‐Garcia et al., 2020; White et al., 1997). Importantly, cytotoxic activity is uniquely regulated in the endometrium, with no effect of menstrual cycle and menopausal status on cytotoxic activity by CD8+ T cells from the endocervix or ectocervix (Rodriguez‐Garcia et al., 2020; White et al., 1997).

**FIGURE 3:**
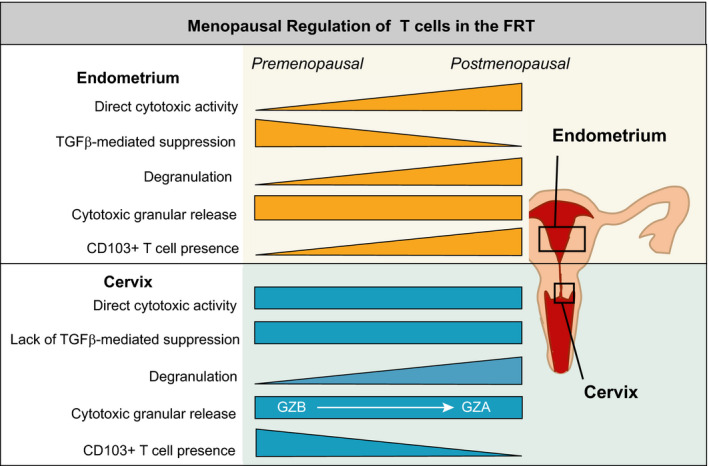
Regulation of CD8+ T‐cell function in the FRT by menopausal status. This diagram indicates key T‐cell functions that are modified after menopause. As indicated by the shape of each triangle, some functions decline while others increase after menopause. Rectangles indicate no change. Effects are shown for the endometrium on the upper part and for the cervix (endocervix and ectocervix) on the lower part of the figure

TGFβ, produced by epithelial cells and fibroblasts (Omwandho et al., 2010; Wira & Rossoll, 2003), has been shown to suppress cytotoxic activity in animal and human experimental models, including the human endometrium (Lee & Rich, 1993; Rodriguez‐Garcia et al., 2020). Interestingly, TGFβ specifically suppressed cytotoxic function of CD8+ T cells from the endometrium but not from the cervix and ectocervix. Susceptibility to endometrial suppression by TGFβ decreased after menopause, highlighting the complex regulation of T cells in the FRT (Rodriguez‐Garcia et al., 2020). Additional major functional changes in CD8+ T cells after menopause include increased degranulation capacity and changes in the profile of granzymes produced, shifting from predominant granzyme B to granzyme A production in response to stimulation (Rodriguez‐Garcia et al., 2020). Granzyme A has proinflammatory properties (Arias et al., 2017; Wensink et al., 2015), and therefore, a modified granzyme A predominant profile after menopause may contribute to increased genital inflammation. Interestingly, while resident and non‐resident T cells have differential cytotoxic capacity (in vitro cytotoxicity and cytotoxic molecule content), both populations equally undergo changes in their granzyme profiles with increased degranulation after menopause (Rodriguez‐Garcia et al., 2020). Changes detected in T cells after menopause are summarized in Figure 3.

Sex hormones modify CD8+ T‐cell cytotoxic activity. Estradiol acts directly on CD8+ T cells, and progesterone indirectly by increasing epithelial cell TGFβ production, to suppress cytotoxicity of endometrial CD8+ T cells (Shen et al., 2021). These findings suggest hormonal suppression of endometrial cytotoxic function, essential for successful implantation and pregnancy. Following menopause, with marked reduction in hormone production, hormonal suppression is removed, and CD8+ T‐cell cytotoxicity may rebound to provide protection throughout the FRT. Potential changes in CD8+ T‐cell cytotoxic activity in the years after menopause are unknown.

## 6 CHANGES IN DISTRIBUTION AND FUNCTION OF DCS AND MACROPHAGES IN THE FRT

In addition to resident T cells, mucosal surfaces contain multiple subsets of resident DCs and macrophages essential for innate immune protection and the induction and maintenance of adaptive immune responses (Schlitzer et al., 2015). The phenotype and function of DCs and macrophages are known to be strongly influenced by the tissue environment (Schlitzer et al., 2015). In the FRT, DCs and macrophages play key roles in reproduction (Dekel et al., 2014; Gnainsky et al., 2015), and their presence in the premenopausal endometrium is regulated by sex hormone (Berbic et al., 2009; Evans & Salamonsen, 2012; Schulke et al., 2008). Therefore, as reproductive function ends, DC and macrophage presence and function in the FRT would be expected to be modified.

Diverse age‐dependent effects have been described for blood DCs, including increased secretion of proinflammatory cytokines, decreased secretion of type I and III IFN, increased responses against self‐antigens, and altered capacity to prime T cells (Agrawal et al., 2017). However, potential age‐dependent changes in DC populations in human tissues, and particularly in the FRT, are not well understood.

**FIGURE 4:**
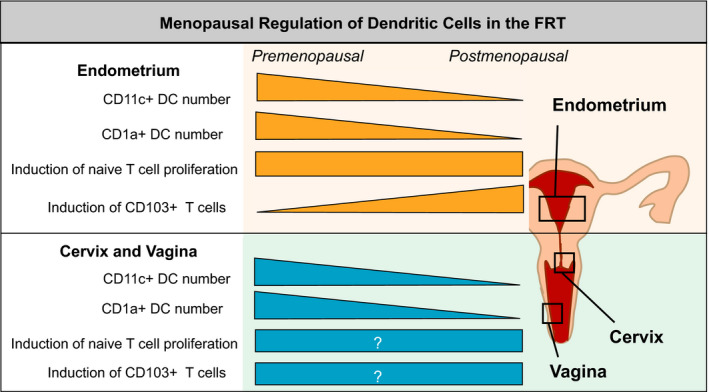
Regulation of DC distribution function in the FRT by menopausal status. This diagram shows key DC functions that are modified after menopause. Specific functions decline or increase after menopause as indicated. Rectangles indicate no change; those containing a question mark (?) indicate that changes are unknown. Effects are shown for the endometrium on the upper part and for the cervix (endocervix and ectocervix) and vagina on the lower part of the figure

Several DC subsets are present throughout the FRT, including CD1c+, CD1a+, and CD14+ DCs. Others have compartmentalized distribution, such as CD103+ DCs, found exclusively in the endometrium, or Langerhans cells and epithelial DCs, found in the vagina (Bertram et al., 2019; Duluc et al., 2013; Hladik et al., 2007; Pena‐Cruz et al., 2018; Rodriguez‐Garcia et al., 2017; Mariani et al., 2002). In the FRT, DCs are found both in the sub‐epithelial compartment and within the epithelium (Iijima et al., 2008; Kaldensjo et al., 2011). We recently demonstrated that as women age, there is a progressive decline in CD11c+ DC number throughout the FRT (Figure 4) (Rodriguez‐Garcia et al., 2018). Decreased presence of CD1a+ antigen presenting cells have also been described in the vaginal mucosa from postmenopausal compared to premenopausal women (Thurman et al., 2017). The decline in DC numbers observed in the FRT contrasts with myeloid DCs in blood and in the intestinal and respiratory mucosae, which remain stable with age (Agrawal, 2017; Granot et al., 2017). Regarding phenotype, a trend toward increased maturation with age has been described in intestinal DCs (Granot et al., 2017), but whether this also applies to FRT DCs remains to be determined. PD‐L1 expression is increased on DCs in endometrium and cervix from postmenopausal compared to premenopausal women (Shen et al., 2016). PD‐L1 increases were specific to DCs and associated with decreased PD‐L1 expression on CD8+ T cells. The potential consequences of these changes on T‐cell activation and peripheral tolerance remain to be determined.

A functional characteristic of DCs is the induction of CD103 expression on naïve CD8+ T cells, suggesting that DCs have the potential to control TRM presence in tissues (Yu et al., 2013). This ability has been demonstrated with human lung and FRT DCs (Duluc et al., 2013; Rodriguez‐Garcia et al., 2018; Yu et al., 2013). Importantly, this function is regulated by menopausal status in the endometrium, with postmenopausal DCs showing enhanced ability to induce CD103 expression on naïve CD8+ T cells when compared to premenopausal DCs (Rodriguez‐Garcia et al., 2018). The mechanism responsible for CD103 upregulation on CD8+ T cells is at least partly related to TGFβ signaling in a contact‐dependent manner (Rodriguez‐Garcia et al., 2018; Yu et al., 2013). Interestingly, this functional modification is highly selective, as it was not associated with changes in FRT DC capacity to induce T‐cell proliferation after menopause (Rodriguez‐Garcia et al., 2018). Potential menopausal regulation of DC function in other FRT compartments remains unknown (Figure 4).

Macrophages constitute 10%–20% of leukocytes in the FRT, with phenotypic differences between upper and lower tract (Givan et al., 1997; Trifonova et al., 2014). In the lower FRT, macrophages express high CD14 levels, while in the endometrium, macrophages express low levels of CD14, with a subset of endometrial macrophages expressing CD163 (Jensen et al., 2012; Quillay et al., 2015; Shen et al., 2009). While it is well known that macrophage numbers increase in the endometrium prior to menstruation (Evans & Salamonsen, 2012), little is known about changes after menopause. An early study reported significant differences in macrophage numbers between pre‐ and postmenopausal women in the fallopian tubes (Safwat et al., 2008), but whether that also applies to the endometrium or lower tract, or whether macrophage functional changes occur is unknown.

Functional changes in DC and macrophage throughout the FRT with aging, and the potential consequences for immune protection and induction of mucosal adaptive responses remain unknown.

## 7 CHANGES IN OTHER FRT CELLS

### 7.1 NK cells

​Natural Killer (NK) cells represent 10–30% of FRT immune cells (Givan et al., 1997; Hunt, 1994; King et al., 1989; Wira et al., 2005). Their numbers in the endometrium change during the menstrual cycle, peaking prior to menstruation (Givan et al., 1997; Hunt, 1994; King et al., 1989; Wira et al., 2005). NK cell phenotype varies within the FRT. In the upper FRT, NK cells are CD56 ^BRIGHT^, CD16‐, and CD94+, whereas in the lower FRT, NK cells are CD56 ^DIM^ CD16+ and CD94‐ (Eriksson et al., 2004; Kopcow et al., 2010; Mselle et al., 2007). FRT NK cells are essential for immune defense against pathogens such as HIV (Mselle et al., 2009; Quillay et al., 2016), control of FRT tumors (Degos et al., 2019), and tissue remodeling for reproduction (Jabrane‐Ferrat, 2019) via their cytotoxic effector functions and cytokine and chemokine secretion.

The extent to which phenotype and numbers of FRT NK cells change in the postmenopausal FRT remains unknown; however, blood NK cells undergo profound changes with aging (Hazeldine & Lord, 2013). Blood NK cell subsets change with age, with decreased CD56^BRIGHT^ cells, increased CD56‐CD16+ cells (Solana et al., 2014), and increased CD57 expression, a marker of differentiated NK cells (Gayoso et al., 2011). The percentage and number of blood CD3‐CD56+ NK cells increases with age (Le Garff‐Tavernier et al., 2010; Lutz et al., 1950, 2005), but is accompanied by reduced proliferation capacity (Solana et al., 1999) suggesting accumulation as a result of increased longevity (Zhang et al., 2007). NK cells in postmenopausal women retain their sensitivity to sex hormones, since estradiol enhances proliferation of blood NK cells (Sho et al., 2017). With respect to cytotoxicity, the effects of age are unclear in that studies report decreased (Hazeldine et al., 2012), increased (Kutza & Murasko, 1994), or no change (Almeida‐Oliveira et al., 2011) in cytotoxic capacity of blood NK cells. NK cells from younger women upregulate IFNγ, MIP‐1α, and IL‐8 to a greater extent than cells from older women (Borrego et al., 1999; Krishnaraj & Bhooma, 1996; Mariani, Meneghetti, et al., 2002; Mariani et al., 2001; Mariani, Pulsatelli, et al., 2002; Solana et al., 1999).

### 7.2 B cells

The density and distribution of B cells varies within the premenopausal FRT, with IgA‐, IgG‐, or IgM‐producing cells predominantly found in the vagina, ectocervix, endocervix, and fallopian tubes, but minimal numbers in the endometrium and ovary (Crowley‐Nowick et al., 1995; Hurlimann et al., 1978; Kelly & Fox, 1979; Kutteh et al., ,^1988^, ^1998^; Rebello et al., 1975; Mariani et al., 2002). In the premenopausal endometrium, during secretory phase, B cells form the central core of endometrial lymphoid aggregates surrounded by CD8+ T cells (Yeaman et al., 1997), but are undetectable in smaller aggregates present during the proliferative phase of the menstrual cycle. In postmenopausal women, aggregates are absent and B cells sparsely distributed throughout endometrial tissue.

Endometrial secretions contain IgG and IgA, with IgG present at higher levels (Schumacher et al., 1980). IgA1 and IgA2 are present in approximately equal proportions (Kutteh et al., 1996). Endometrial secretion of IgA peaks shortly before ovulation (Kutteh et al., 1996; Schumacher et al., 1973, 1977, 1980), while stromal IgA peaks at ovulation (Kelly & Fox, 1979). IgA and IgG levels in cervical mucus also vary with stage of the menstrual cycle and are lowest at midcycle (Schumacher et al., 1973). However, in other studies, IgA and IgG were suppressed during secretory phase (Keller et al., 2007). There was no difference in IgG and IgA levels in cervico‐vaginal secretions between premenopausal, postmenopausal, and pregnant women (Jilanti & Isliker, 1977). In postmenopausal vaginal secretions, IgG and IgA levels were reduced by twofold and 15‐fold, respectively, following hysterectomy (Jilanti & Isliker, 1977), demonstrating significant endometrial contributions to FRT IgG and IgA levels.

Immunoglobulins present in FRT secretions are essential components of immune protection. IgG and IgA neutralize incoming pathogens and prevent their entry into target cells (Lamm et al., ,^1978^, ^1995^; Nedrud et al., 1987). For example, anti‐HIV IgM reduces infection of DCs in cervical‐vaginal explant tissues (Devito et al., 2018) while levels of anti‐HIV gp160 IgG antibodies in human CVL samples correlate with anti‐HIV activity and reduce HIV infection of target cells *in vitro* (Ghosh et al., 2010). Immunoglobulins also bind to mucus in FRT secretions via mucin proteins and trap pathogens within it. Anti‐HSV‐1 IgG, via its Fc component, traps HSV‐1 in human cervical‐vaginal mucus thus preventing contact with target cells (Wang et al., 2014). Intriguingly, while both IgA and IgG bind to cervical mucus, only IgG binds to cervical‐vaginal mucus (Fahrbach et al., 2013). Vaccination at peripheral sites elicits antibody‐mediated mucosal protection in the FRT. For example, vaccination against HPV16 in premenopausal women leads to increased titers of anti‐HPV IgG in cervical secretions that varies with menstrual cycle stage (Nardelli‐Haefliger et al., 2003). How aging affects the contribution of immunoglobulin‐mediated protection in the FRT is unknown.

## 8 CONCLUSIONS

The mucosal immune system in the human FRT has uniquely evolved to meet the challenges of an external environment as well as support new life. Across multiple anatomical compartments, mucosal immunity is precisely regulated to protect against sexually transmitted pathogens while accommodating allogeneic spermatozoa and an immunologically distinct semi‐allogeneic fetus. While much is known about the mucosal immune system in the FRT during the reproductive years, little is known about the changes that occur after menopause as women age. Limited studies into innate and adaptive immune functions in the FRT following menopause indicate that immune protection by epithelial cells, stromal fibroblasts, T cells, and DC in the FRT are compromised, with limited compensation. Much remains to be learned about the impact of age following menopause on immune protection in the FRT. Understanding the impact of age on mucosal immune protection in the FRT is crucial given the challenges women face in terms of urogenital infections, exposure to sexually transmitted pathogens, and gynecological cancers that threaten the lives of women worldwide. This review emphasizes the need for additional studies to provide a foundation for the development of age‐appropriate therapeutic interventions that increase protection in older women, the fastest growing segment of the population in developed countries.

## CONFLICT OF INTEREST

The authors declare no conflict of interest.

## AUTHOR CONTRIBUTIONS

MRG and CRW wrote the introduction section; MVP wrote epithelial cell, fibroblast, and other cells sections; MRG, CRW, and ZS wrote the T‐cell section; MRG wrote the DC section. All authors edited and approved the manuscript.
